# Healing Time of Skin Ulcers in Homecare Residents in the Province of Reggio Emilia, Northern Italy

**DOI:** 10.3390/life12121989

**Published:** 2022-11-28

**Authors:** Inga Iamandii, Abram Beatrice Kouassi, Davide Simonazzi, Cristina Marchesi, Marco Vinceti, Tommaso Filippini

**Affiliations:** 1 CREAGEN—Environmental, Genetic and Nutritional Epidemiology Research Center, Section of Public Health, Department of Biomedical, Metabolic and Neural Sciences, University of Modena and Reggio Emilia, 41125 Modena, Italy; 2 Cardiology Unit, Department of Specialty Medicine, Azienda USL-IRCCS di Reggio Emilia, 42123 Reggio Emilia, Italy; 3 Department of Primary Care, Azienda USL-IRCCS di Reggio Emilia, 42122 Reggio Emilia, Italy; 4 Head Office, Azienda USL-IRCCS di Reggio Emilia, 42122 Reggio Emilia, Italy; 5 Department of Epidemiology, Boston University School of Public Health, Boston, MA 02118, USA; 6 School of Public Health, University of California Berkeley, Berkeley, CA 94704, USA

**Keywords:** skin ulcer, healing time, homecare setting, primary care, nursing care

## Abstract

The growing phenomenon of skin ulcers represents an important health problem; therefore, we conducted a pilot study to evaluate the ulcer healing time among adult subjects followed by the Home Nursing Service of the AUSL-IRCCS of Reggio Emilia, Northern Italy, and diagnosed with at least one skin ulcer during the period of January–August 2020. We recruited 138 subjects (45.5% men) with a mean age of 86.1 years. The subjects presented with 232 ulcers, of which 76.7% were pressure ulcers (60.1% were stage II), 18.1% were vascular ulcers, and 4.7% were diabetic foot ulcers. Ulcer management required only one weekly access for the majority of subjects, with a recovery frequency of 53.6% at the end of the observation period. The median ulcer healing time was 3.6 months and was shorter in women (2.6 months) than men (5.1 months), with an increasing trend according to the number of ulcers and the severity of pressure ulcers for vascular and diabetic foot ulcers. In conclusion, this is the first study carried out in an Italian population describing the distribution and characteristics of homecare residents with skin ulcers and highlighting the factors influencing the healing time and as consequence the duration of nursing care.

## 1. Introduction

Skin ulcers are defined as a disruption in the physiological structure and function of the skin, which may also affect the underlying soft tissue structures [1]. Acute ulcers normally proceed through an orderly and timely reparative process that results in sustained restoration of anatomic and functional integrity [1], whereas chronic ulcers fail to progress through an orderly healing process within an amount of time that normally should be sufficient to re-establish anatomic and functional integrity [1,2,3]. Nevertheless, there is no universally accepted definition of chronic ulcers because there is no consensus with respect to the duration required to define an ulcer as chronic [4,5], although in some studies, the recovery time varies from 4 weeks up to more than 3 months [2,5,6], and there is no consensus on the physical description of the wound and the timeframe for wound healing [7]. Skin ulcers are often but not systematically classified, as a result of their underlying cause, into acute, such as unhealed surgical wounds, burns, traumatic wounds, etc.; chronic, such as arterial leg ulcers, venous leg ulcers, mixed leg ulcers, diabetic foot ulcers, and classic pressure ulcers [4,8,9,10,11,12,13]; and skin lesions found in patients at the end of life or in advanced stages of mostly incurable diseases, i.e., deep tissue lesions, unavoidable pressure injuries, skin failure, ischemic wounds, cancer, etc. [14,15,16].

The phenomenon of skin ulcers represents an important healthcare issue because it is a serious burden for individuals and their caregivers, widely affecting their quality of life [6,8,17,18]. Similarly, the management of skin ulcers is an important task for the healthcare system, requiring considerable use of human and economic resources [6,9], accounting for 2–4% of the healthcare budgets in developed countries [3,17,18,19]. Skin ulcers have been described as a “Silent Epidemic” [3,8], given that 1–2% of the population in the industrialized world will be affected at least once during their lifetime [3,19,20]. In addition, the burden of skin ulcers is rapidly increasing, owing to the continuous improvement of sociosanitary conditions, population aging, and increased incidence of chronic diseases, especially type 2 diabetes [3,6,17,18,19,21]. It is estimated that approximately 70–90% of ulcer care in Europe is conducted within the community, the majority of such care being delivered by community nurses [8,19]. Studies carried out in Europe and North America have shown that 25–35% of all individuals with ulcers in the community are treated in their own home [19]. The most frequently treated ulcers treated in the community setting are acute/surgical ulcers, pressure ulcers, and leg ulcers [17,19]. However, owing to heterogeneous terminology in the literature and variability in study designs and approaches in assessing prevalence and incidence [7], data vary, and it is challenging to obtain a reliable estimate of this phenomenon, resulting in an underreported health issue, with varying frequency of estimation both within and across care settings [3,7].

The main objective of our study was to evaluate the healing time of skin ulcers of subjects followed by the Home Nursing Service of the AUSL-IRCCS of Reggio Emilia. An additional objective was to analyze the intrinsic and extrinsic characteristics of subjects that can positively or negatively affect ulcer healing.

## 2. Materials and Methods

Following approval by the AVEN “Area Vasta Emilia Nord” Ethics Committee (no. 1063/2020 on 20 October 2020), we conducted a pilot study in the province of Reggio Emilia, Northern Italy. We included in the study all the subjects in care at the Home Nursing Service (“Servizio Infermieristico Domiciliare”) of the Primary Healthcare Centre “Casa della Salute Nord” of the AUSL-IRCCS of Reggio Emilia who had received a diagnosis of at least one skin ulcer from 1 January to 31 August 2020.

The Home Nursing Service is a part of the Italian Health Care System that offers assistance at home to people who are not self-sufficient and in fragile conditions, making them unable to attend health facilities. The organization comprises homecare nurses who, in the organizing of the care plan, collaborate with general practitioners, medical specialists, social services, and voluntary associations with the aim of stabilizing the clinical picture, limiting functional decline, and improving the quality of life of subjects and their families [22]. The 8-home nursing team of the Primary Healthcare Centre “Casa della Salute Nord” of the AUSL-IRCCS of Reggio Emilia is available for all inhabitants from the areas of the northern part of the province (approximately 70,000 people).

For our study, we extracted data from local health authority database ADI-Web, which contains information about subjects in the care of the Home Nursing Service, as well as their caregiver, which was routinely collected by nurses during personal interviews and examinations. The eligible subjects were adults with at least one type of skin ulcer diagnosed from 1 January to 31 August 2020, including pressure ulcers, vascular ulcers, and diabetic foot ulcers. Among this population, we included in our study a sample for which all the following information was available in the database: demographic characteristics (sex and age), characteristics of skin ulcers (number; type; site; stage; date of onset; and date of healing, considered by nurses as complete tissue repair and a lack of need for further treatment), degree of dependence of the subject (fully dependent, partially dependent, or autonomous), availability and type of caregiver present at home, and the number of weekly accesses by the Home Nursing Service. Furthermore, for our analysis, we also extracted information available from the service activation sheet (i.e., containing the date of first activation of the homecare service due to new diagnosis of skin ulcer; care needs; and date of closure of the service for recovery, death, or hospitalization), and in the nursing deliveries in order to obtain full details regarding skin ulcers. We collected data through anonymized individual codes, with data presented in aggregated form only.

The continuous variables were expressed as mean and standard deviation, whereas the categorical variables were expressed as absolute frequency and percentage. For data analysis, to investigate healing time, we used a survival analysis model using the Kaplan-Meier estimator. In detail, we calculated the survival function related to the duration of homecare considering the subjects in charge until one of the following outcomes (whichever occurred first): recovery, hospitalization, or death of the subject; end of homecare; or the date of termination of the study (31 August 2020). Subjects with truncated observation times because they were still in charge at the end of the observation period, deceased, or hospitalized were considered “censored” at the date of the end of the study or at the date of death/rehospitalization. We computed survival functions according to pressure ulcer severity, the presence of vascular ulcers or diabetic foot ulcers, frequency of home accesses by nurses, degree of dependence of patients, and number of skin ulcers. Moreover, we calculated the median time of care for the entire examined sample and for the subgroups of interest, such as ulcer type and stage, degree of dependence, and number of weekly accesses by the nursing staff. For all statistical analyses, we used Stata software (v17.0 2021, StataCorp LCC, College Station, TX, USA).

## 3. Results

Among the approximately 400 subjects under the care of the Home Nursing Service from 1 January to 31 August 2020, 145 patients were eligible for inclusion in this study, as they had been diagnosed with at least one skin ulcer during the study period. We included 138 subjects with ages ranging from 43 to 104 years and a mean of 86.1 years (Table 1), the relevant information for whom was registered in the ADI-web database. One-third of study participants were men (N = 49), and two-thirds were women (N = 89), with women having a higher average age: 83.2 (SD: 9.8) years and 87.8 (SD: 8.9) years, respectively.

Among all study participants, 107 (77.5%) subjects had at least one pressure ulcer, 36 (26.1%) had at least one vascular ulcer, and 10 (7.2%) had at least one diabetic foot ulcer. Considering the ulcer with the highest severity among subjects was pressure ulcers, we found that 13 (12.1%), 69 (64.5%), 8 (7.5%), 8 (7.5%), and 9 (8.4%) subjects had at least one stage I, stage II, stage III, unstageable, and stage IV ulcer, respectively. We also found that 11 subjects who had a pressure ulcer also had a vascular ulcer, and 2 had a diabetic foot ulcer, whereas, among those without a pressure ulcer, 23 had only a vascular ulcer, and 6 had only a diabetic foot ulcer (Table 2).

In terms of the number of ulcers (Table 2), the subjects presented a total of 232 skin ulcers, of which 178 (76.7%) were pressure ulcers, 42 (18.1%) were vascular ulcers, and 11 (4.7%) were diabetic foot ulcers. Therefore, the type of ulcers that nurses managed most frequently pressure ulcers. Furthermore, 82 (59.4%) subjects had only one ulcer, 30 (21.7%) had two ulcers, 17 (12.3%) had three, 7 (5.1%) had four, 1 (0.7%) had five, and 1 (0.7%) had six ulcers. The most frequent locations for pressure ulcers were the sacrum (N = 70, 39.3%), gluteus (N = 17, 9.6%), and heel (N = 35, 19.7%), whereas for vascular ulcers and diabetic foot ulcers, the most common location was the foot (N = 31, 73.8% and N = 11, 100% respectively), as shown in Table 2.

**Figure 1:**
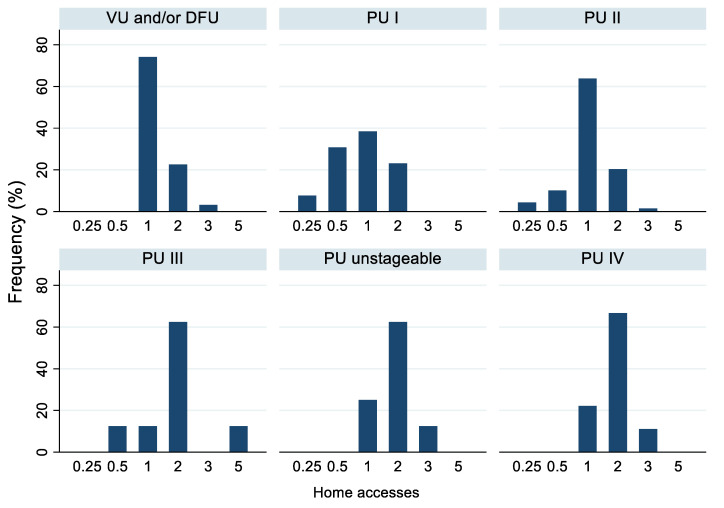
Frequency of home accesses (times/week) according to type and stage of ulcers. Abbreviations: PU pressure ulcer; DFU, diabetic foot ulcer; VU vascular ulcer. Number of home accesses: 0.25 = one access in 4 weeks, 0.5 = one access in 2 weeks, 1 = one access/week, 2 = two accesses/week, 3 = three accesses/week, 5 = five accesses/week.

**Figure 2:**
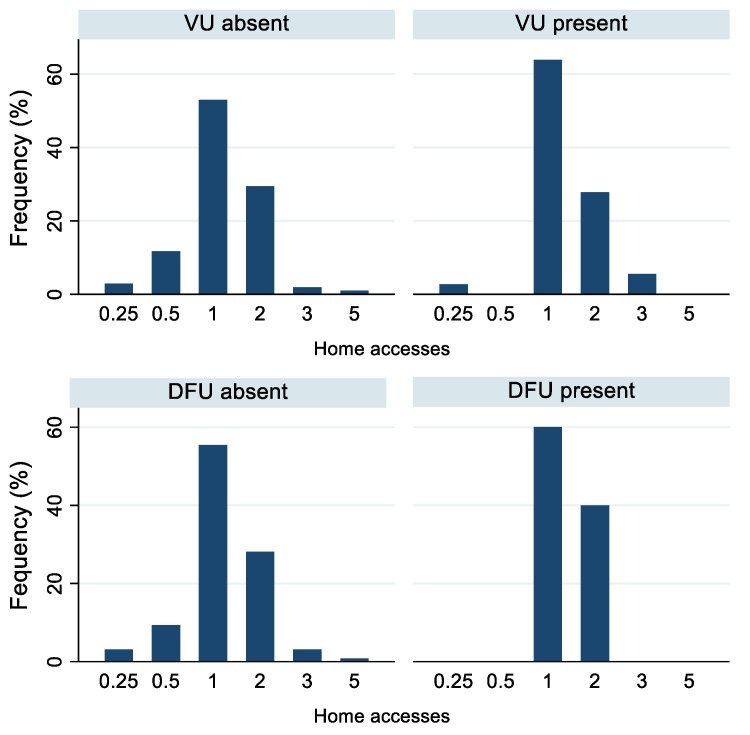
Frequency of home accesses (times/week) according to presence or absence of vascular and diabetic foot ulcers (subjects with VU or DFU vs. subjects without VU or DFU). Abbreviations: DFU, diabetic foot ulcer; VU, vascular ulcer. Number of home accesses: 0.25 = one access in 4 weeks, 0.5 = one access in 2 weeks, 1 = one access/week, 2 = two accesses/week, 3 = three accesses/week, 5 = five accesses/week.

**Figure 3:**
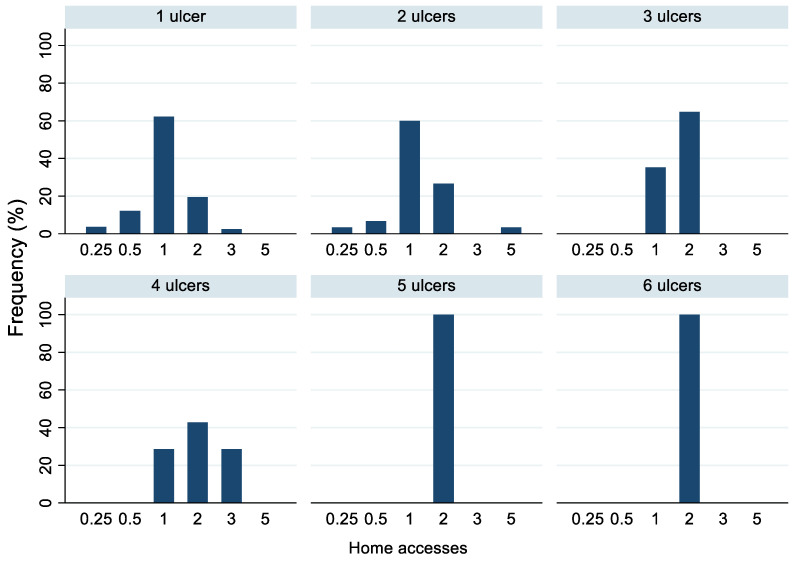
Frequency of home accesses (times/week) according to number of lesions. Number of home accesses: 0.25 = one access in 4 weeks, 0.5 = one access in 2 weeks, 1 = one access/week, 2 = two accesses/week, 3 = three accesses/week, 5 = five accesses/week. A total of 82 subjects had one ulcer, 30 had two ulcers, 17 had three ulcers, 7 had four ulcers, 1 had five ulcers, and 1 had six ulcers.

In terms of the number of home accesses, ulcer management required only one weekly access in 56% of cases, two in 29%, and one access every 15 days in 8.7% of cases (Table 3). The frequency of accesses varied in relation to the type and stage of ulcer presented by the subject: twice a month or weekly for stage I; mainly weekly for stage II, vascular, and diabetic foot ulcers; and biweekly for stage III, IV, and unstageable ulcers (Figure 1 and Figure 2). In addition, subjects with 1–2 ulcers were visited mostly once a week, whereas those with ≥3 ulcers were accessed mainly twice a week (Figure 3). Moreover, we observed that both women and men were mainly treated once a week, and 14.3% of subjects with family and non-family caregivers were visited once every 2 weeks, whereas those living alone were visited mainly once or twice a week (Table 3).

Considering the degree of dependence, most of the subjects in our study were totally dependent (N = 89, 64.5%), with a predominance of women compared to men. Moreover, subjects with vascular injuries and diabetic foot injuries were dependent in 53% and 60% of cases, respectively, and the degree of dependence increased with severity of the pressure injury (Table 4).

At the end of the study period, 74 patients (53.6%) had recovered, 32 (23.2%) were deceased before their ulcers could fully heal, 8 (5.8%) were hospitalized, and 24 (17.4%) were still being followed by the service (Table 5). Of those fully recovered, two-thirds were women, 58.1% were totally dependent, and 64.9% received nursing care once a week. Furthermore, most stage I, II, and vascular ulcers healed (53.8%, 60.9%, and 61.1%, respectively), whereas only 37.5% of stage III, 25% of unstageable ulcers, 11.1% of stage IV, and 20% of diabetic foot ulcers had a positive outcome (Table 5).

**Figure 4:**
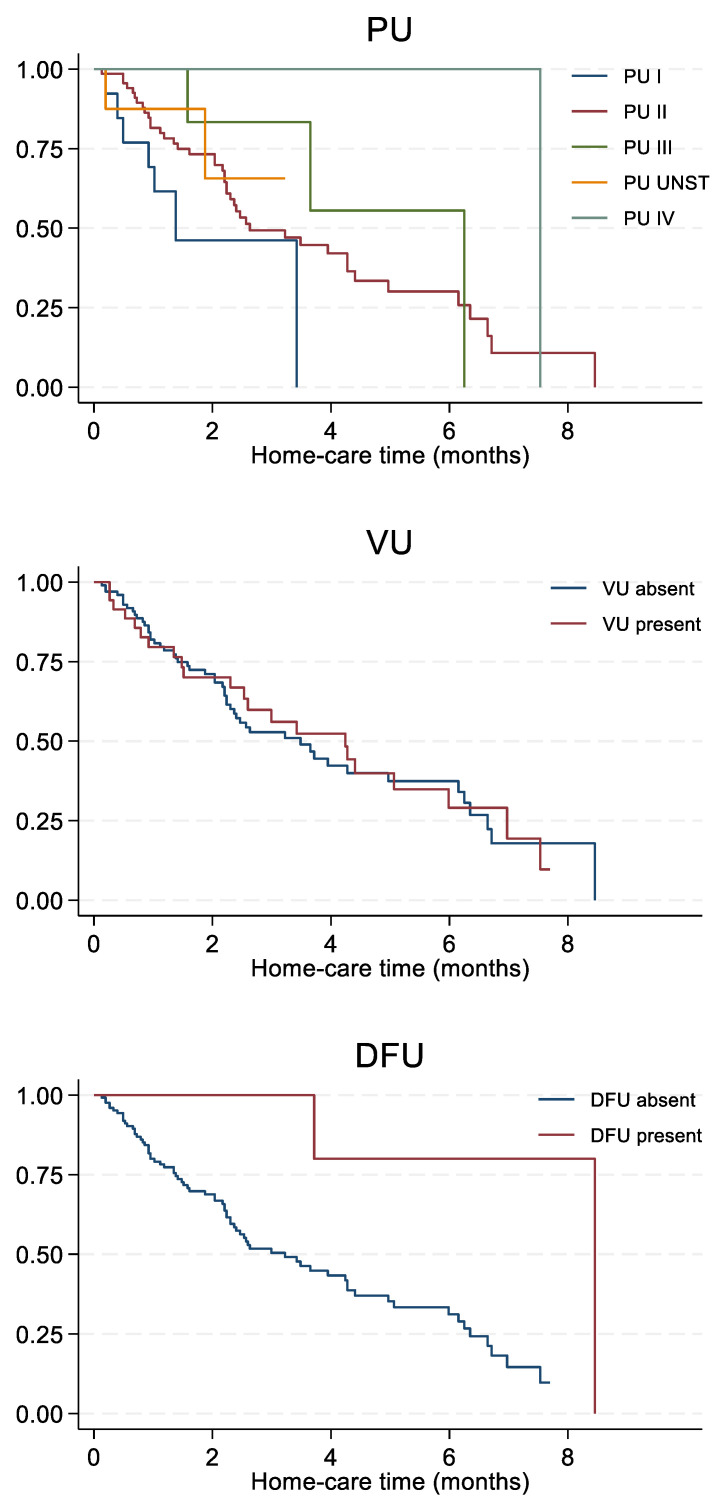
Homecare time (months) according to type and stage of ulcers. Abbreviations: PU, pressure ulcer; DFU, diabetic foot ulcer; VU, vascular ulcer.

In our study, the median ulcer healing time for the entire sample was 3.6 months and was generally shorter in women (2.6 months) than men (5.1 months). Figure 4 shows an increasing trend of healing time according to ulcer severity, with a healing time of 1.4 months for stage I, 2.6 months for stage II, 6.3 months for stage III, and 7.5 months for stage IV and unstageable ulcers. The median period of care was slightly higher for subjects with vascular ulcers compared to those without (4.2 vs. 3.5 months, respectively), whereas it was substantially higher for those with diabetic foot ulcers compared to those without diabetic foot ulcers (8.5 vs. 3.2 months, respectively) (Figure 4).

**Figure 5:**
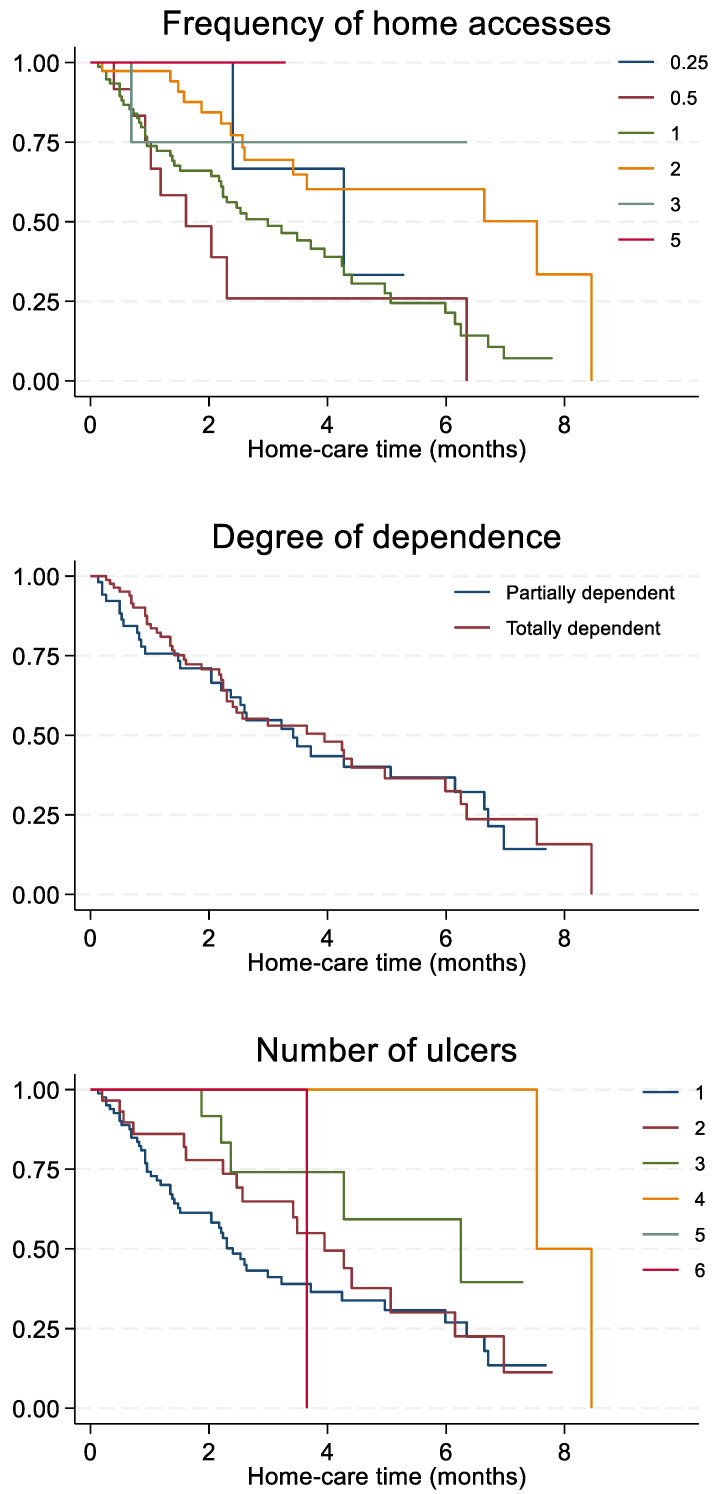
Homecare time (months) according to frequency of home accesses, degree of dependence, and number of ulcers. Number of home accesses: 0.25 = one access in 4 weeks, 0.5 = one access in 2 weeks, 1 = one access/week, 2 = two accesses/week, 3 = three accesses/week, 5 = five accesses/week.

Furthermore, those who were followed once a month had a longer healing time (4.3 months) than those who were accessed once per week or once every 15 days (2.9 and 1.6 months, respectively), although still shorter than those followed twice a week (7.5 months) (Figure 5). In addition, we found that partially dependent subjects recovered slightly faster than totally dependent subjects (3.4 vs. 3.9 months, respectively). Lastly, homecare time increased concurrently with the number of ulcers, starting with 2.4 months and 3.9 months for those with one and two ulcers, respectively, and ending with 6.3 months and 7.5 months for those with three and four ulcers, respectively.

## 4. Discussion

We aimed to investigate skin ulcer healing time among subjects treated in a homecare setting by conducting a pilot study in the province of Reggio Emilia, Northern Italy. Data analysis showed that the 138 subjects followed by the Home Nursing Service had an average age of 86 years, which was higher than that reported in most studies on subjects with skin ulcers in homecare settings, ranging from 68 to 83 years [23,24,25,26,27,28,29,30,31,32,33,34]. In our sample, we observed that women were more affected than men, and our findings are consistent with most of the literature [23,24,25,26,27,28,30,31,32,33,34,35]. Moreover, in our study, the average age of women presenting with at least one ulcer (87 years) was higher than that of men (83 years).

Considering the type of skin ulcers managed at home, we found that the most frequent type was pressure ulcers (77%), followed by vascular and diabetic foot ulcers, in accordance with the reports of most previous studies [23,24,27,28,31,32,33,34,36]; for example, pressure ulcers reached a frequency of almost 95% in the Italian SILP report [36]. Conversely, one study [31] reported that vascular ulcers were most frequent, followed by pressure ulcers and diabetic foot ulcers. Furthermore, our results show that the most common locations of pressure ulcers were the sacrum, heel, and gluteus, whereas in other studies [25,26,33,35,37,38], the sacrum, heel, and coccyx were reported as the most affected anatomical sites. Moreover, stage II pressure ulcers were the most frequent, in line with most of the literature [23,25,26,35,37] and in contrast with Lee et al. [39] and Queiroz et al. [40], who reported stages 3–4 as the most frequent ulcers. Finally, most of our subjects had one ulcer, as in other studies [25,26,27,28,39].

In our study, ulcer management required only one weekly access in 56% of cases, in contrast with a study by Rodrigues et al. [39] regarding chronic wounds in homecare, in which more than 50% of subjects were treated two to four times per week. In addition, our results show that the frequency of home accesses was affected by the ulcer type and stage (i.e., mainly weekly for vascular, diabetic foot, and stage I–II pressure ulcers and biweekly for the other stages of pressure ulcers), as well as the number of ulcers (i.e., mostly once a week for 1–2 ulcers and twice a week for ≥3 ulcers).

More than half of the subjects recovered during the study period, followed by those who were hospitalized or deceased. A similar pattern was reported by Zarchi et al. [24], Volpe et al. [33], and Sankaran et al. [41], although the second studied chronic ulcers and the third studied PU in a palliative homecare setting. On the contrary, Artico et al. [26] reported opposite behavior, with the majority of the subjects remaining unhealed or deceased, although the study setting was different, i.e., a palliative homecare center. Of those fully recovered in our study, the majority fell into the following categories: women, totally dependent, and treated mostly one a week. Furthermore, we found that the type and stage of ulcers had an impact on the outcome; most subjects with vascular and pressure stage I–II ulcers healed, whereas those with diabetic foot and more severe pressure ulcers had a lower rate of positive outcomes.

In our study, the Home Nursing Service followed subjects for a median of 3.6 months, which is a longer period than in the study by Teot et al. [42] (2.3 months), similar to that used by Pieper et al. [28] (3.8 months), and a shorter period than those reported in the studies regarding chronic wounds in homecare settings conducted by Rodrigues et al. [32] (12 months), Lanau-Roig et al. [31] (21 months), and Panfil et al. [34] (6.7 years). The differences in healing time of skin ulcers in homecare could be explained by the fact that the intrinsic type of assessment relies on subjective evaluation of healthcare personnel [4,5,43,44]. Therefore, nurses involved in our study may have had difficulties in determining when an ulcer could be considered effectively healed, reporting staging data within the nursing records, implementing diverse treatment types, and assessing the presence of other environmental and socioeconomic factors [24].

Few studies have been published in the literature investigating the healing time of skin ulcers in homecare settings, as most studies were conducted in other care settings. We found that women were more likely to experience a shorter healing time, as previously reported by Artico et al. [26] but not by Pieper et al. [28], who reported that sex had no effect. In addition, we observed an increasing trend according to pressure ulcer severity, which was also observed by Artico et al. [26], who reported an average healing time of 21.2 days for pressure ulcers. Furthermore, patients healed slower if they had vascular ulcers or diabetic foot ulcers, in contrast to the results reported by Lanau-Roig et al. [31], who observed that chronic pressure ulcers healed more slowly than chronic vascular ulcers (average of 41.97 months and 13.2 months, respectively). Lastly, in our study, subjects recovered faster if they were partially dependent, if they had fewer ulcers, and if they were visited by nurses ≤ 1/week, although the latter seems to be associated with the frequency of home accesses according to ulcer type and stage.

Our study is subject to some limitations. First, it was not possible to classify vascular ulcers according to the Leriche-Fontaine or Rutherford classification [45], nor the revised CEAP classification [46], and it was not possible to classify diabetic foot ulcers according to the Wagner or University of Texas classification [47], nor to make a clear distinction between venous and arterial ulcers because these data were lacking within the ulcer classification sheet or within the nursing deliveries, as well as within the ADI-web system, in which vascular injuries were classified generically as stage I, II, or III. This may explain some of the differences relative to previous studies findings, limiting the comparability of our findings. Second, it was not possible to assess the characteristics of the bed of the ulcers, except for pressure ulcer staging, nor the worsening of the ulcers, if any. In addition, we did not assess the type of therapeutic intervention (dressings). With respect to subject health status, we had limited information on comorbidities, as there was no mandatory data collection form concerning the subjects’ medical history within the ADI-Web system, and the nurses did not always include this information in the deliveries at the first activation visit of the homecare service. Despite the limitations, to the best of our knowledge, this is the first study carried out in an Italian population assessing the healing time of skin ulcers in a homecare setting outside of palliative care, providing an overview of the characteristics and determinants of the duration of homecare service.

## 5. Conclusions

A longer duration of nursing homecare is required for subjects with diabetic foot, vascular, and stage ≥ III pressure ulcers, although stage II ulcers occur more frequently. Proper characterization of ulcers and systematic recording of information on subjects’ general health status in the nursing record could significantly improve the quality and the organization of homecare service for skin ulcers.

## Figures and Tables

**Table 1:** Population characteristics (age values reported in years).

Characteristics	Men	Women	All
Subjects with ulcers N (%)	49 (35.5%)	89 (64.5%)	138 (100%)
Age, mean (SD)	83.2 (9.8)	87.8 (8.9)	86.1 (9.4)

**Table 2:** Characteristics of skin lesions divided by type and stage.

	Pressure Ulcers						Other Types of Ulcers		All Ulcers
	S. I	S. II ± I	S. III ± I/II	S. IV ± I/II/III	Unstageable ± I/II/III	All Stages	VU	DFU	
**Subjects with ulcers** **N (%)**	13 (9.4%)	69 (50%)	8 (5.8%)	9 (6.5%)	8 (5.8%)	107 (77.5%)	36 (26.1%) ^1^	10 (7.2%) ^2^	138 (100%)
**Age,** **mean (SD)**	87.7 (6.9)	86.6 (10.3)	85.4 (5.1)	92.8 (7.6)	87.6 (4.3)	87.2 (9.1)	85.4 (9.4)	77.6 (9.6)	86.1 (9.4)
**Number N (%) and location of ulcers**									
**Pelvis and hip**	**18 (48.6%)**	**58 (54.2%)**	**8 (53.3%)**	**5 (5.6%)**	**2 (20%)**	**91 (51.1%)**	−	−	**91 (39.2%)**
Sacrum	14 (37.8%)	45 (42.1%)	6 (40%)	5 (5.6%)	−	70 (39.3%)	−	−	70 (30.2%)
Trochanter	1 (2.7%)	4 (3.7%)	1 (6.7%)	−	2 (20%)	8 (4.5%)	−	−	8 (3.5%)
Hip	1 (2.7%)	2 (1.9%)	−	−	−	3 (1.7%)	−	−	3 (1.3%)
Groin	1 (2.7%)	1 (0.9%)	−	−	−	2 (1.2%)	−	−	2 (0.9%)
Ischium	1 (2.7%)	6 (5.6%)	−	−	−	7 (3.9%)	−	−	7 (3%)
Other	−	−	1 (6.7%)	−	−	−	−	−	1 (0.4%)
**Foot**	**12 (32.4%)**	**26 (24.3%)**	**5 (33.3%)**	**4 (4.4%)**	**7 (70%)**	**54 (30.3%)**	**31 (73.8%)**	**11 (100%)**	**96 (41.4%)**
Heel	8 (21.6%)	17 (15.9%)	3 (20%)	1 (11.1%)	6 (60%)	35 (19.7%)	3 (7.1%)	2 (18.2%)	40 (17.2%)
Big toe	2 (5.4%)	1 (0.9%)	1 (6.7%)	1 (11.1%)	1 (10%)	6 (3.4%)	6 (14.3%)	5 (45.5%)	17 (7.3%)
Malleolus	1 (2.7%)	6 (5.6%)	1 (6.7%)	−	−	8 (4.5%)	4 (9.5%)	−	12 (5.2%)
Other toe	−	1 (0.9%)	−	1 (11.1%)	−	2 (1.2%)	6 (14.3%)	2 (18.2%)	10 (4.3%)
Other	1 (2.7%)	1 (0.9%)	−	1 (11.1%)	−	3 (1.7%)	12 (28.6%)	2 (18.2%)	17 (7.3%)
**Lower limb**	**6 (16.2%)**	**16 (14.9%)**	**2 (13.3%)**	−	−	**21 (11.8%)**	**11 (26.2%)**	−	**32 (13.8%)**
Gluteus	4 (14.8%)	13 (12.1%)	−	−	−	17 (9.6%)	−	−	17 (7.3%)
Knee	1 (2.7%)	1 (0.9%)	1 (6.7%)	−	−	2 (1.2%)	−	−	2 (0.9%)
Thigh	−	1 (0.9%)	−	−	−	1 (0.6%)	−	−	1 (0.4%)
Calf	−	−	1 (6.7%)	−	−	1 (0.6%)	−	−	1 (0.4%)
Other	1 (2.7%)	1 (0.9%)	−	−	−	−	11 (26.2%)	−	13 (5.6%)
**Trunk and back**	**1 (2.7%)**	**7 (6.5%)**	−	−	**1 (10%)**	**9 (5.1%)**	−	−	**9 (3.9%)**
**Total**	37 (100%) (15.9%) ^†^	**107 (100%) (46.1%) ^†^**	**15 (100%) (6.5%) ^†^**	**9 (100%)** **(3.9%) ^†^**	**10 (100%) (4.3%) ^†^**	**178 (100%)** **(76.7%) ^†^**	**42 (100%)** **(18.1%) ^†^**	**11 (100%)** **(4.7%) ^†^**	**232 (100%)** **(100%) ^†^**

^1^ A total of 11 with pressure ulcers and 23 without pressure ulcers; ^2^ two with pressure ulcers and eight without pressure ulcers; ^†^ fraction (%) of the ulcer number according to type and stage, with reference to the total number of ulcers. Abbreviations: DFU, diabetic foot ulcer; N, number; PU pressure ulcer; S, stage; SD, standard deviation; VU, vascular ulcer.

**Table 3:** Number of home accesses divided by frequency and characteristics of subjects.

	1 in 4 Weeks	1 in 2 Weeks	1 in a Week	2 in a Week	3 in a Week	5 in a Week
**Ulcer type and stage**						
PU all stages	4 (3.7%)	12 (11.2%)	54 (50.5%)	33 (30.8%)	3 (2.8%)	1 (0.9%)
PU I	1 (7.7%)	4 (30.8%)	5 (38.5%)	3 (23.1%)	−	−
PU II	3 (4.3%)	7 (10.1%)	44 (63.8%)	14 (20.3%)	1 (1.5%)	−
PU III	−	1 (12.5%)	1 (12.5%)	5 (62.5%)	−	1 (12.5%)
PU IV	−	−	2 (22.2%)	6 (66.7%)	1 (11.1%)	−
PU unstageable	−	−	2 (25.5%)	5 (62.5%)	1 (12.5%)	−
DFU	−	−	6 (60%)	4 (40%)	−	−
VU	1 (2.8%)	−	23 (63.9%)	10 (27.8%)	2 (5.6%)	−
**Sex**						
Men	2 (4.1%)	2 (4.1%)	23 (57.1%)	18 (36.7%)	3 (6.1%)	1 (2%)
Women	2 (2.2%)	10 (11.2%)	54 (60.7%)	22 (24.7%)	1 (1.1%)	−
**Caregiver**						
Relative	3 (4.8%)	5 (7.9%)	32 (50.8%)	21 (33.3%)	2 (3.2%)	−
Relative and professional	−	4 (14.3%)	16 (57.1%)	7 (25%)	−	1 (3.6%)
Not reported	1 (5.3%)	1 (5.3%)	11 (57.9%)	5 (26.3%)	1 (5.3%)	−
Professional	−	2 (8%)	16 (64%)	6 (24%)	1 (4%)	−
Living alone	−	−	2 (66.7%)	1 (33.3%)	−	−
**Degree of dependence**						
Partially dependent	2 (3.8%)	1 (1.9%)	33 (63.5%)	15 (28.8%)	1 (1.9%)	−
Totally dependent	2 (2.3%)	11 (12.8%)	44 (51.2%)	25 (29.1%)	3 (3.5%)	1 (1.2%)
**Total, N (%)**	**4 (2.9%)**	**12 (8.7%)**	**77 (55.8%)**	**40 (29%)**	**4 (2.9%)**	**1 (0.7%)**

Abbreviations: PU, pressure ulcer; DFU, diabetic foot ulcer; VU, vascular ulcer.

**Table 4:** Degree of dependence. Values are number (N) and percentage (%) of subjects.

Characteristics	Partially Dependent	Totally Dependent
**Sex**		
Men	21 (40.4%)	31 (59.6%)
Women	28 (32.6%)	58 (67.4%)
**Type and stage of ulcer**		
PU I	4 (30.8%)	9 (69.2%)
PU II	26 (37.7%)	43 (62.3%)
PU III	1 (12.5%)	7 (87.5%)
PU IV	−	9 (100%)
PU unstageable	1 (12.5%)	7 (87.5%)
**PU all stages**	**32 (29.9%)**	**75 (70.1%)**
VU	19 (52.8%)	17 (47.2%)
DFU	6 (60%)	4 (40%)
**Total, N (%)**	**49 (35.5%)**	**89 (64.5%)**

Abbreviations: PU, pressure ulcer; DFU, diabetic foot ulcer; VU, vascular ulcer.

**Table 5:** Distribution of outcomes. Values are number (N) and percentage (%) of subjects.

	Still in Care	Recovered	Deceased	Hospitalized
**Sex**				
Men	8 (16.3%)	24 (49%) *(32.4%)* ^‡^	15 (30.6%)	2 (4.1%)
Women	16 (17.8%)	50 (56.2%) *(67.6%)* ^‡^	17 (19.1%)	6 (6.7%)
**Type and stage of ulcer**				
PU all stages	17 (15.8%)	55 (51.4%) *(74.3%)* ^‡^	30 (28.1%)	5 (4.7%)
PU I	3 (23.1%)	7 (53.8%) *(9.5%)* ^‡^	3 (23.1%)	−
PU II	9 (13%)	42 (60.9%) *(56.8%)* ^‡^	17 (24.6%)	1 (1.5%)
PU III	1 (12.5%)	3 (37.5%) *(4.1%)* ^‡^	3 (37.5%)	1 (12.5%)
PU IV	3 (33.3%)	1 (11.1%) (1.4%) ^‡^	3 (33.3%)	2 (22.2%)
PU unstageable	1 (12.5%)	2 (25%) *(2.7%)* ^‡^	4 (50%)	1 (12.5%)
VU	5 (13.9%)	22 (61.1%) *(29.7%)* ^‡^	6 (16.7%)	3 (8.3%)
DFU	3 (30%)	2 (20%) *(2.7%)* ^‡^	1 (10%)	4 (40%)
**Degree of dependence**				
Partially dependent	13 (25%)	31 (59.6%) *(41.9%)* ^‡^	6 (11.5%)	2 (3.8%)
Totally dependent	11 (12.8%)	43 (50%) *(58.1%)* ^‡^	26 (30.2%)	6 (7%)
**Number of home accesses**				
1 in 4 weeks	2 (50%)	2 (50%) *(2.7%)* ^‡^	−	−
1 in 2 weeks	−	9 (75%) *(12.2%)* ^‡^	2 (16.7%)	1 (8.3%)
1 in a week	10 (13%)	48 (62.3%) *(64.9%)* ^‡^	14 (18.2%)	5 (6.5%)
2 in a week	11 (27.5%)	14 (35%) *(18.9%)* ^‡^	13 (32.5%)	2 (5%)
3 in a week	1 (25%)	1 (25%) *(1.4%)* ^‡^	2 (50%)	−
5 in a week	−	−	1 (100%)	−
**Total, N (%)**	**24 (17.4%)**	**74 (53.6%) *(100%)*** ^‡^	**32 (23.2%)**	**8 (5.8%)**

^‡^ Percentages (%) of the subjects healed according to sex, type and stage of ulcers, degree of dependence, and number of home accesses, with reference to the total number of healed subjects. Abbreviations: PU, pressure ulcer; DFU, diabetic foot ulcer; VU, vascular ulcer.

## Data Availability

The data presented in this study are available upon reasonable request from the corresponding author. The data are not publicly available due to privacy issues.
